# Supplementary material for: Longitudinal hierarchical Bayesian models of covariate effects on airway and alveolar nitric oxide
Source: Sci Rep. 2023 Apr 1;13:5346. doi: 10.1038/s41598-023-31774-7 (PMC10067946; doi:10.1038/s41598-023-31774-7)
Supplement: Supplementary file 1 — Supplementary Information. [file 41598_2023_31774_MOESM1_ESM.docx]

Supplementary materials for

**Title:** **Longitudinal Hierarchical Bayesian models of covariate effects on airway and alveolar nitric oxide**

**Authors:** Jingying Weng^1^, Noa Molshatzki^1^, Paul Marjoram^1^, W. James Gauderman^1^, Frank D. Gilliland^1^, Sandrah P. Eckel^1*^

## **Summary of two-stage methods used to estimate associations of covariates with longitudinally assessed NO parameters**

##### L_TS_NLS: Stage I consists of N (participants) x M (visits) separate NLS [10] models, each NLS model fit to the typically small multiple flow FeNO dataset at that visit using a model of the form where *k* indexes the repeated maneuvers for a given participant/visit (typically 9 in the CHS):

$log({FeNO}_{k}) =log \left( C_{aw}+\left( C_{A}-C_{aw} \right)\times e^{-\frac{D_{aw}}{{flow}_{k}}} \right)+\varepsilon_{k}$

##### L_TS_HMA: Similarly, Stage I consists of N x M separate HMA [19, 20] models. See the original HMA publications for the 3^rd^ order approximation based HMA algorithm.

##### L_TS_NLME: Stage I consists of a single longitudinal nonlinear least square mixed effect (NLME) model, an extension of the approach using N x M separate NLS models. In the longitudinal NLME, we specified participant-level (*i*) and visit-level (*j*) random intercepts for each NO parameter. At each level, these random effects follow a multivariate normal distribution, allowing for correlation of NO parameters. We implemented NLME using the nlme package in R.

$\log\left( \mathrm{FeNO}_{ijk} \right) = log\left( \text{exp}\left( {{logC}_{aw}}_{ij} \right)+\left( {C_{A}}_{i}-\text{exp}\left( {{logC}_{aw}}_{ij} \right) \right)\times e^{-{\text{exp}\left( {{logD}_{aw}}_{ij} \right)}/{{flow}_{ijk}}} \right)+ \varepsilon_{ijk}$

##### L_TS_HB: Stage I consists of a single longitudinal Hierarchical Bayesian (HB) analog of the longitudinal NLME model, implemented using JAGS similar to the U-HB cross-sectional model in our previous publication [12], but with no covariate X and a partitioning of variance in NO parameters at the participant- and visit-levels through specification of variance-covariance matrices, the population mean of the NO parameters, and the measurement error in Stage I.

All the above TS approaches use the same LMM approach in Stage II, where Stage I estimates of the three NO parameters for participant *i* at visit *j* (${\hat{C_{A}}}_{ij}$, ${\hat{{logC}_{aw}}}_{ij}$, and ${\hat{{logD}_{aw}}}_{ij}$) are treated as known values and used as the outcomes in three separate Stage II linear mixed models relating each NO parameter to a covariate X, each with a participant-level random intercept (e.g., $\alpha_{{C_{A}}_{i}}$ for the $C_{A}$ model):

$\begin{aligned} {\hat{C_{A}}}_{ij}=\beta_{0_{CA}}+\alpha_{{C_{A}}_{i}}+ \beta_{C_{A}}X_{ij}+\varepsilon_{{C_{A}}_{ij}} \\ {\hat{{logC}_{aw}}}_{ij}=\beta_{0_{{logC}_{aw}}}+\alpha_{{{logC}_{aw}}_{i}}+ \beta_{{logC}_{aw}}X_{ij}+\varepsilon_{{{logC}_{aw}}_{ij}} \\ {\hat{{logD}_{aw}}}_{ij}=\beta_{0_{{logD}_{aw}}}+\alpha_{{{logD}_{aw}}_{i}}+ \beta_{{logD}_{aw}}X_{ij}+\varepsilon_{{{logD}_{aw}}_{ij}} \end{aligned}$

We now show a number of further details of results of our simulation study. We begin by exploring convergence rates across a range of methods and scenarios.

**Supplementary Table 1a：Number of simulation study datasets (out of 100) with successful convergence for each method and scenario.**

| Method | Scenario 1 | | | Scenario 2 | | | Scenario 3 | | | Scenario 4 | | |
| --- | --- | --- | --- | --- | --- | --- | --- | --- | --- | --- | --- | --- |
|  | $\beta_{Ca}$ = $\beta_{logCaw}$ = $\beta_{logDaw}$ | | | $\beta_{logCaw}$ = $\beta_{logDaw}$ = 0 | | | $\beta_{Ca}$ = $\beta_{logDaw}$ = 0 | | | $\beta_{Ca}$ = $\beta_{logCaw}$ = 0 | | |
|  |  |  |  | $\beta_{Ca}$ | | | $\beta_{logCaw}$ | | | $\beta_{logDaw}$ | | |
|  | 0.01 | 0.05 | 0.1 | 0.01 | 0.05 | 0.10 | 0.01 | 0.05 | 0.10 | 0.01 | 0.05 | 0.10 |
| L_U_HB | 95 | 100 | 89 | 89 | 97 | 98 | 91 | 88 | 95 | 90 | 97 | 92 |
| L_TS_HB | 100 | 99 | 99 | 97 | 99 | 100 | 98 | 100 | 100 | 100 | 100 | 99 |
| L_U_NLME | 54 | 55 | 49 | 49 | 48 | 48 | 46 | 45 | 47 | 56 | 55 | 56 |
| L_TS_NLME | 96 | 96 | 93 | 96 | 95 | 93 | 95 | 96 | 92 | 93 | 92 | 96 |
| L_TS_HMA | 100 | 100 | 100 | 100 | 99 | 100 | 100 | 100 | 100 | 100 | 100 | 99 |
| L_TS_NLS | 100 | 100 | 100 | 100 | 100 | 100 | 100 | 100 | 100 | 100 | 100 | 100 |
| All methods converged | 53 | 54 | 41 | 45 | 45 | 47 | 41 | 39 | 44 | 51 | 54 | 50 |

**Supplementary Table 1b：Average Stage I convergence failure rates* for select two-stage methods: L_TS_HMA and L_TS_NLS. ^[[1]](#footnote-1)^**

| Method | Scenario 1 | | | Scenario 2 | | | Scenario 3 | | | Scenario 4 | | |
| --- | --- | --- | --- | --- | --- | --- | --- | --- | --- | --- | --- | --- |
|  | $\beta_{Ca}$ = $\beta_{logCaw}$ = $\beta_{logDaw}$ | | | $\beta_{logCaw}$ = $\beta_{logDaw}$ = 0 | | | $\beta_{Ca}$ = $\beta_{logDaw}$ = 0 | | | $\beta_{Ca}$ = $\beta_{logCaw}$ = 0 | | |
|  |  |  |  | $\beta_{Ca}$ | | | $\beta_{logCaw}$ | | | $\beta_{logDaw}$ | | |
|  | 0.01 | 0.05 | 0.1 | 0.01 | 0.05 | 0.10 | 0.01 | 0.05 | 0.10 | 0.01 | 0.05 | 0.10 |
| L_TS_HMA | 0.2 | 0.2 | 0.3 | 0.2 | 0.2 | 0.3 | 0.3 | 0.3 | 0.2 | 0.3 | 0.3 | 0.2 |
| L_TS_NLS | 435.3 | 435.6 | 439.3 | 432.4 | 434.5 | 435.1 | 433.5 | 433.5 | 433.6 | 438.2 | 434.2 | 436.3 |

#

# Supplementary Table 2: Average Computation time for each method and simulation scenario.

Average computation times on a high-performance computing platform (3 CPU, 8GB memory) for a single simulated dataset (500 participants, 3 visits each, 8 maneuvers per visit) were: 29 hours for L_U_HB, 24 hours for L_TS_HBL, 14.7 minutes for L_U_NLME, 11.6 minutes for L_TS_NLME, 4.7 seconds for L_TS_HMA and 6.1 seconds for L_TS_NLS. However, the computation times differed slightly across scenarios. Table 2 summarizes the computation time of each scenario for all methods.

| Scenario | Scenario 1 | | | Scenario 2 | | | Scenario 3 | | | Scenario 4 | | | overall |
| --- | --- | --- | --- | --- | --- | --- | --- | --- | --- | --- | --- | --- | --- |
|  | $\beta_{Ca}$ = $\beta_{logCaw}$ = $\beta_{logDaw}$ | | | $\beta_{logCaw}$ = $\beta_{logDaw}$ = 0 | | | $\beta_{Ca}$ = $\beta_{logDaw}$ = 0 | | | $\beta_{Ca}$ = $\beta_{logCaw}$ = 0 | | |  |
|  |  |  |  | $\beta_{Ca}$ | | | $\beta_{logCaw}$ | | | $\beta_{logDaw}$ | | |  |
|  | 0.01 | 0.05 | 0.1 | 0.01 | 0.05 | 0.10 | 0.01 | 0.05 | 0.10 | 0.01 | 0.05 | 0.10 |  |
| L_U_HB  (hours） | 28 | 32.3 | 28.4 | 33.4 | 31.1 | 31.5 | 31.6 | 30.2 | 30.2 | 30.4 | 28 | 30 |  |
| L_TS_HB  (hours) | 25.4 | 22.7 | 23.7 | 22.6 | 23 | 21.8 | 23 | 21.7 | 23.3 | 21.3 | 22.7 | 22.4 |  |
| L_U_NLME  (minutes) | 18.5 | 13.7 | 11.7 | 15.2 | 17.3 | 14.7 | 16.5 | 14.6 | 14.4 | 12.3 | 18.2 | 15.3 |  |
| L_TS_NLME  (minutes) | 12.4 | 11.4 | 11 | 12.2 | 12.1 | 12.6 | 12.7 | 11.1 | 11.9 | 12.9 | 13.4 | 12.5 |  |
| L_TS_HMA  (seconds) | 4.9 | 4.6 | 4.7 | 4.7 | 4.4 | 4.5 | 4.4 | 4.5 | 4.5 | 4.5 | 4.5 | 4.6 |  |
| L_TS_NLS  (seconds) | 6.1 | 6.2 | 5.8 | 6.2 | 5.8 | 5.9 | 5.7 | 5.9 | 6.1 | 5.8 | 6 | 5.8 |  |

#

#

# Supplementary Figure 1: Extensive comparison of method performance in Simulation Scenarios with subplots for the different parameters

The performance of methods were compared in terms of bias, 95% CI length and 95% CI coverage for coefficients, populational means, participant level standard deviations, participant level correlations and visit level correlations of NO parameters ($\beta_{Ca}$: black square, $\beta_{logCaw}$: red circle, $\beta_{logDaw}$: blue triangle), replicated at 3 different effect sizes ($\beta$ = 0.01, 0.05 or 0.1). Power and type I error rates for coefficients. These results are shown below.

## A: Scenario 1

| A1: Estimated Associations β |
| --- |
| 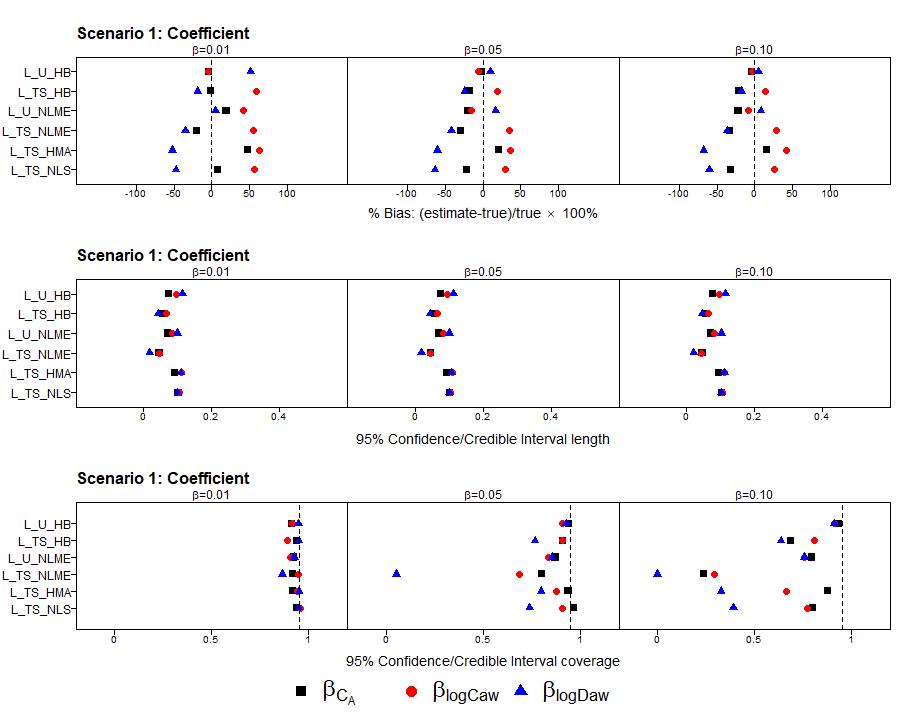 |
| 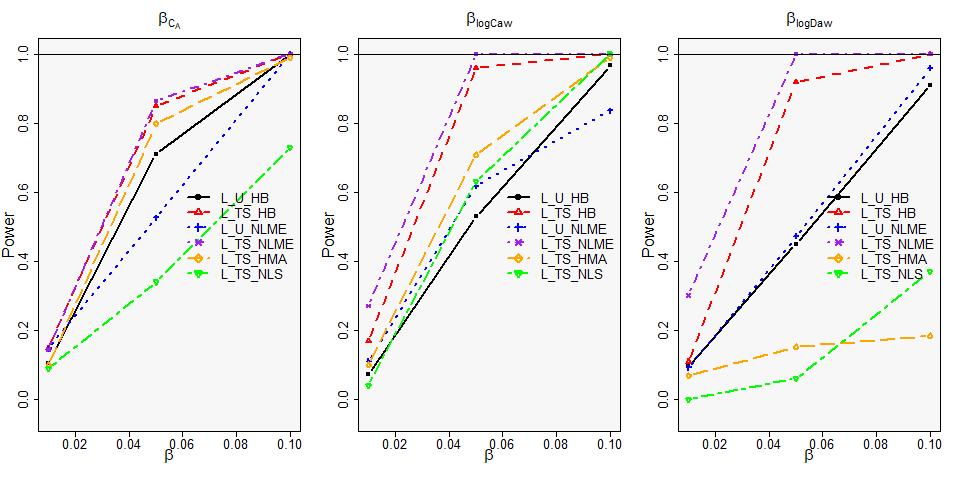 |
|  |
|  |

| A2: Population level mean ɑ |
| --- |
|  |


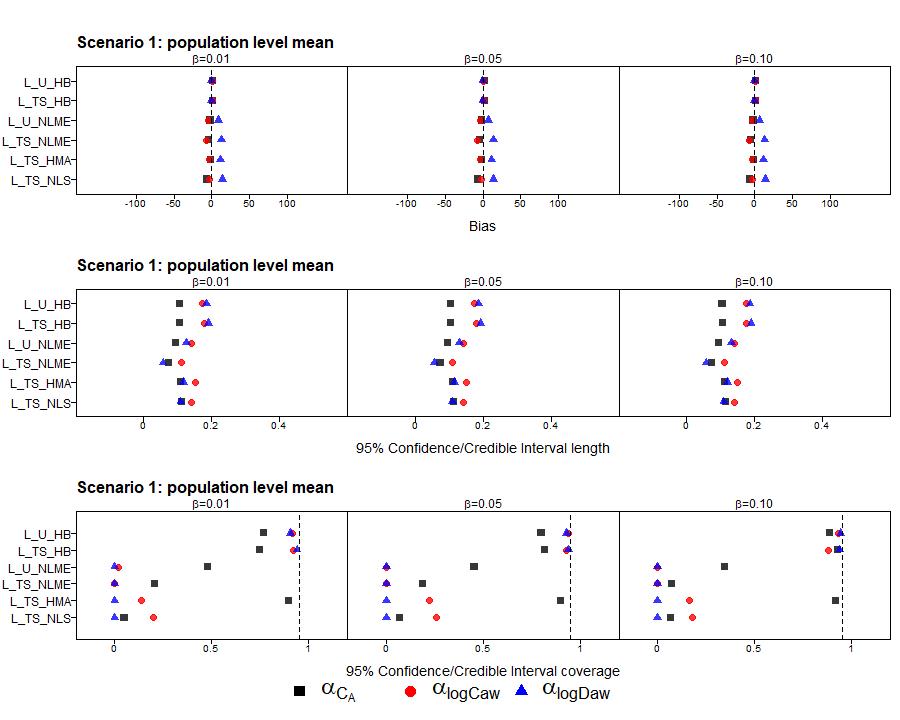


| A3: Participant level standard deviations τ |
| --- |
| 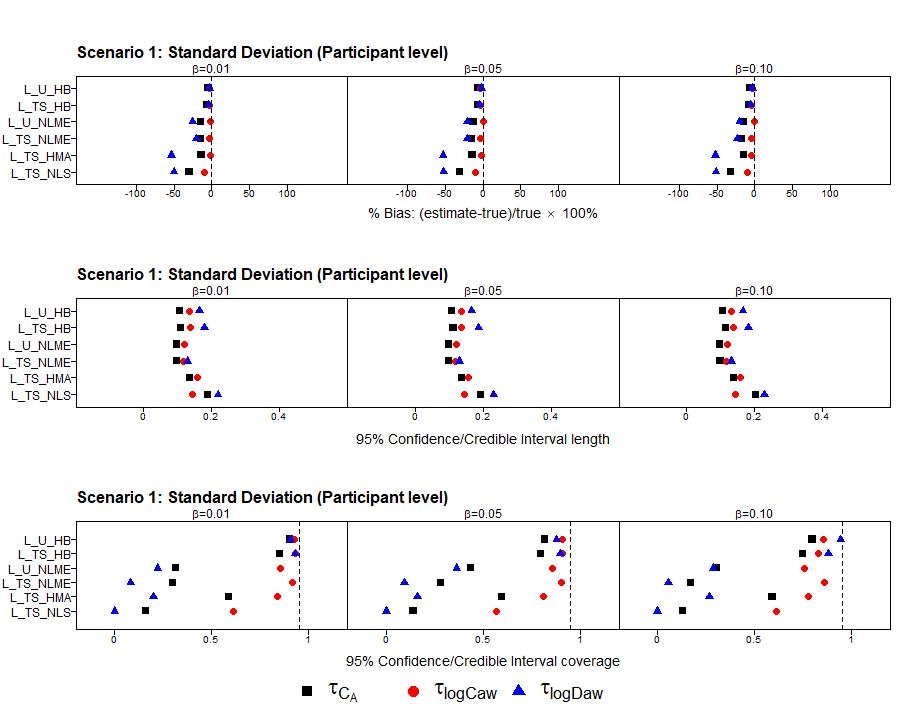 |
|  |
|  |

| A4: Participant level correlations ⍴ |
| --- |
| 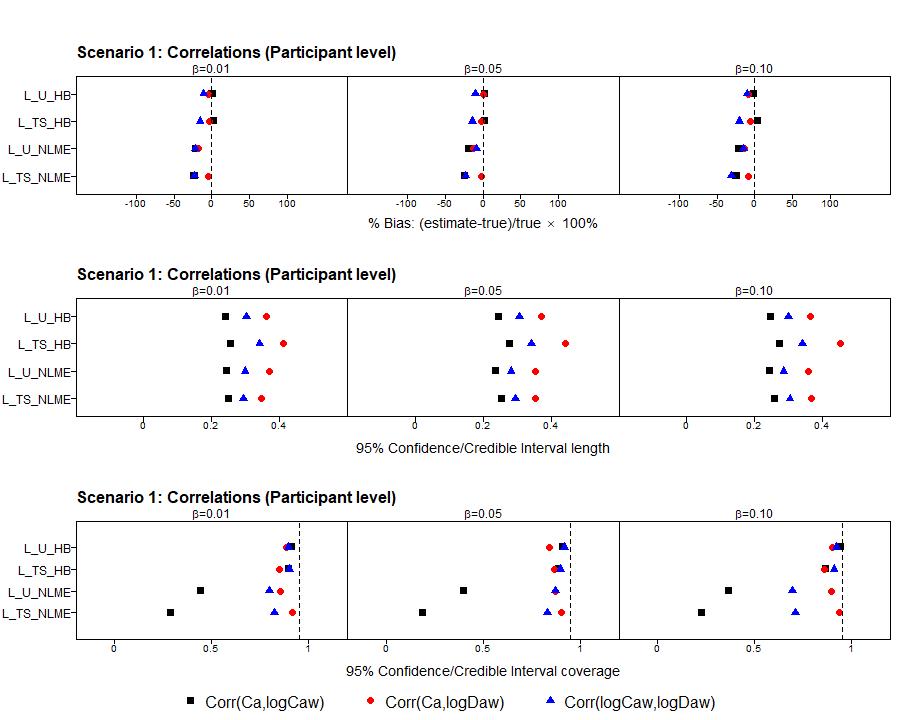 |

| A5: Visit level standard deviations σ |
| --- |
| 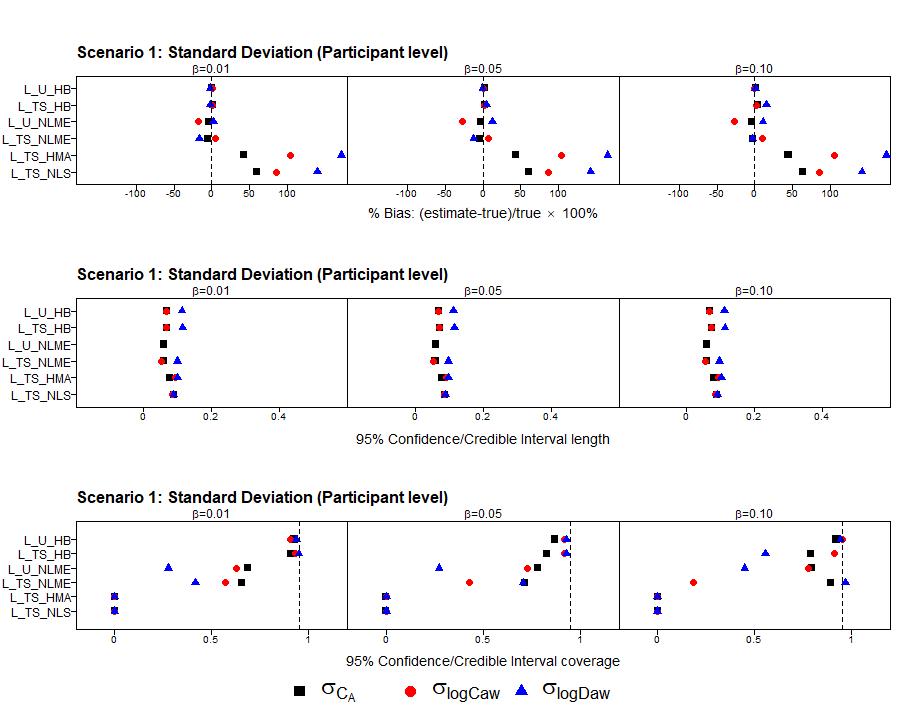 |

## B: Scenario 2

| B1: Estimated Associations β (Bias instead of % Bias) |
| --- |
| 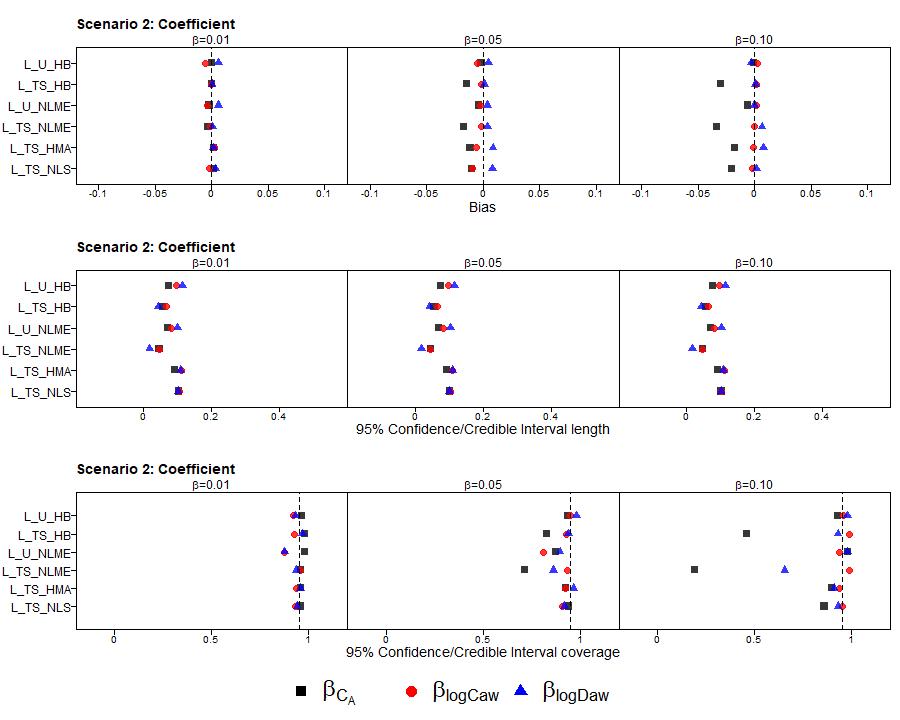 |
|  |
| 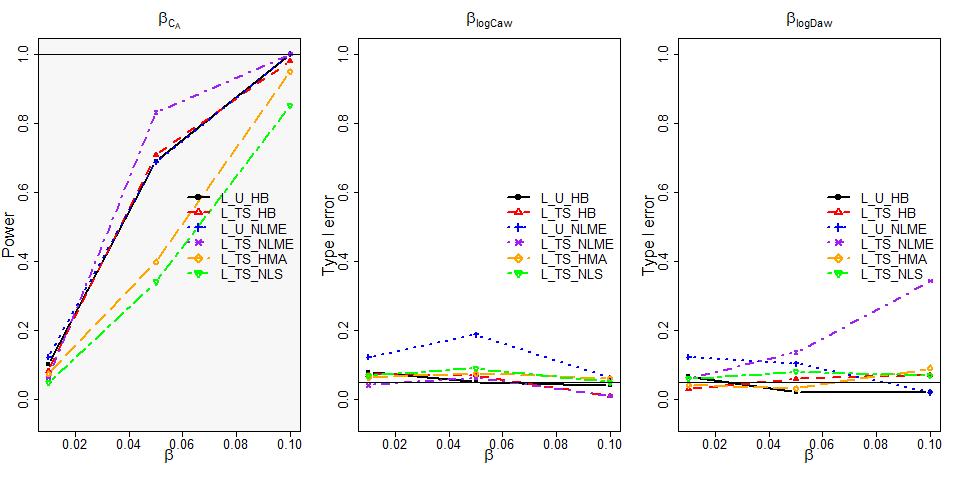 |

| B2: Population level mean ɑ |
| --- |
| 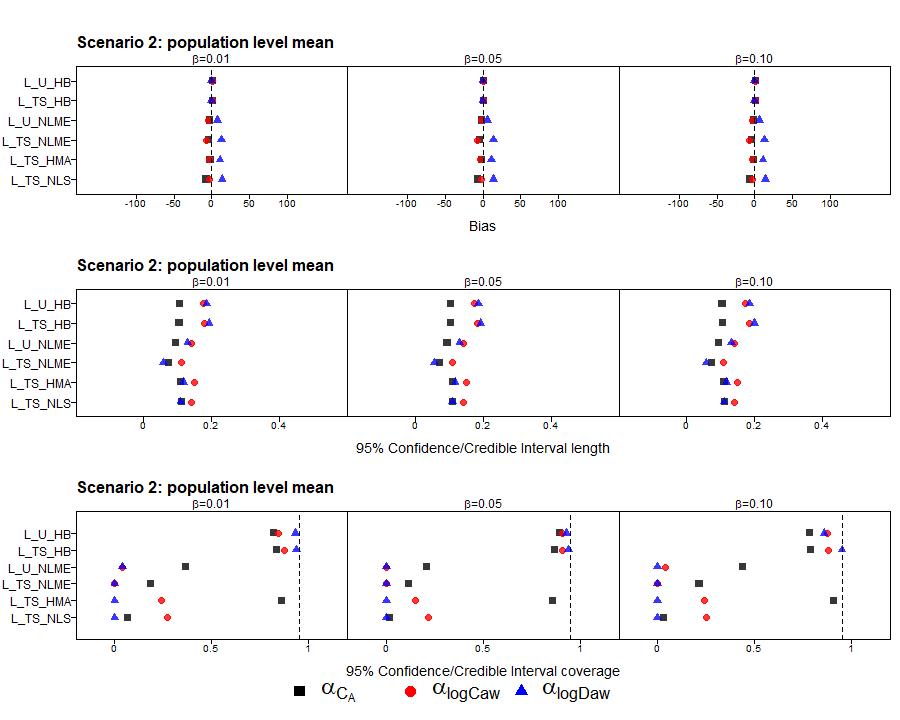 |

| B3: Participant level standard deviations τ |
| --- |
| 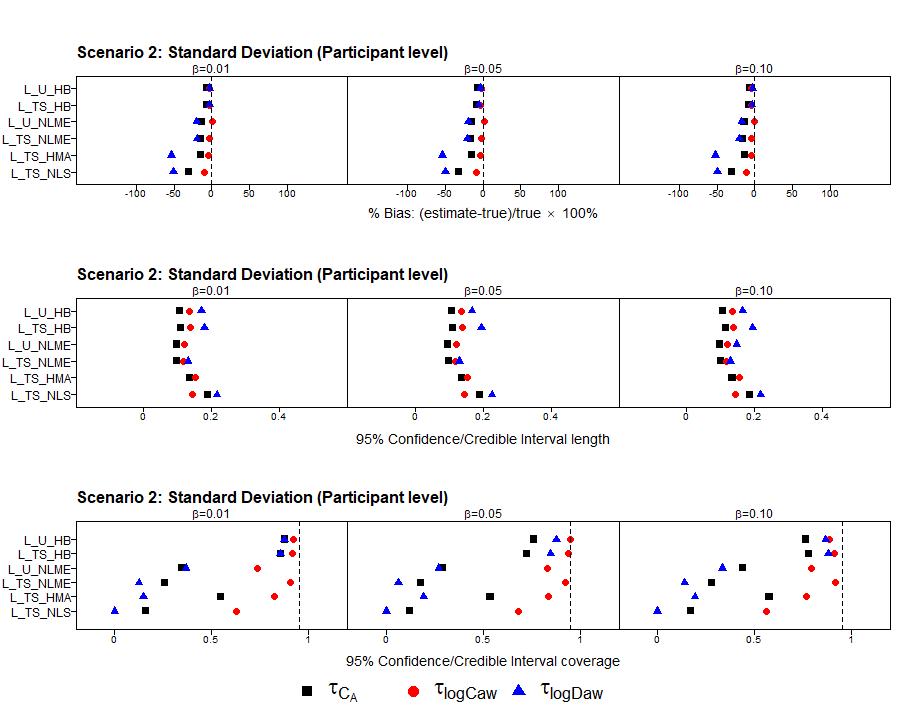 |

| B4: Participant level correlations ⍴ |
| --- |
| 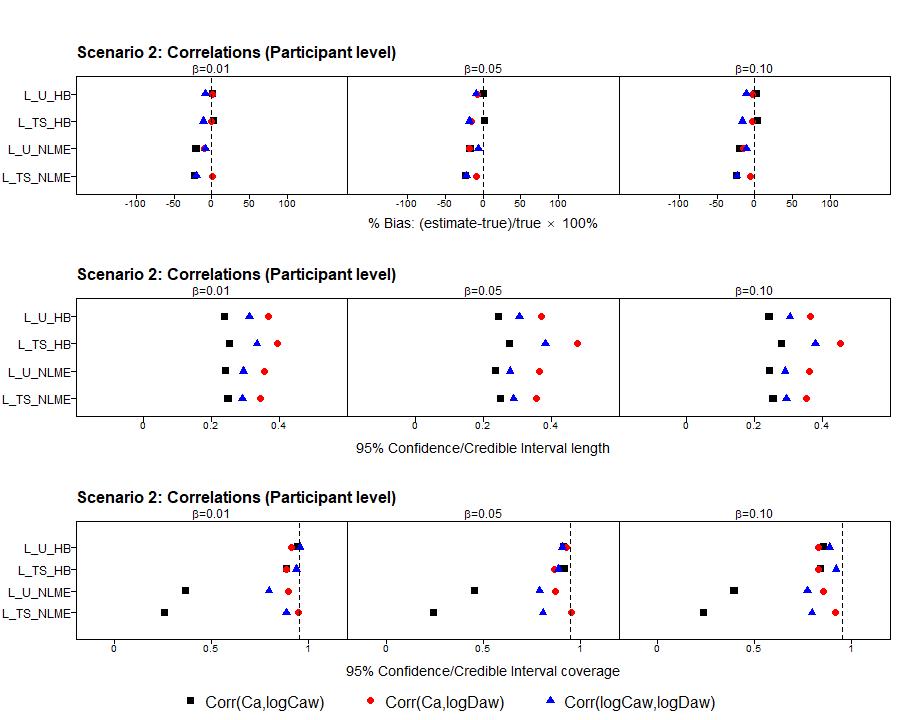 |

| B5: Visit level standard deviations σ |
| --- |
| 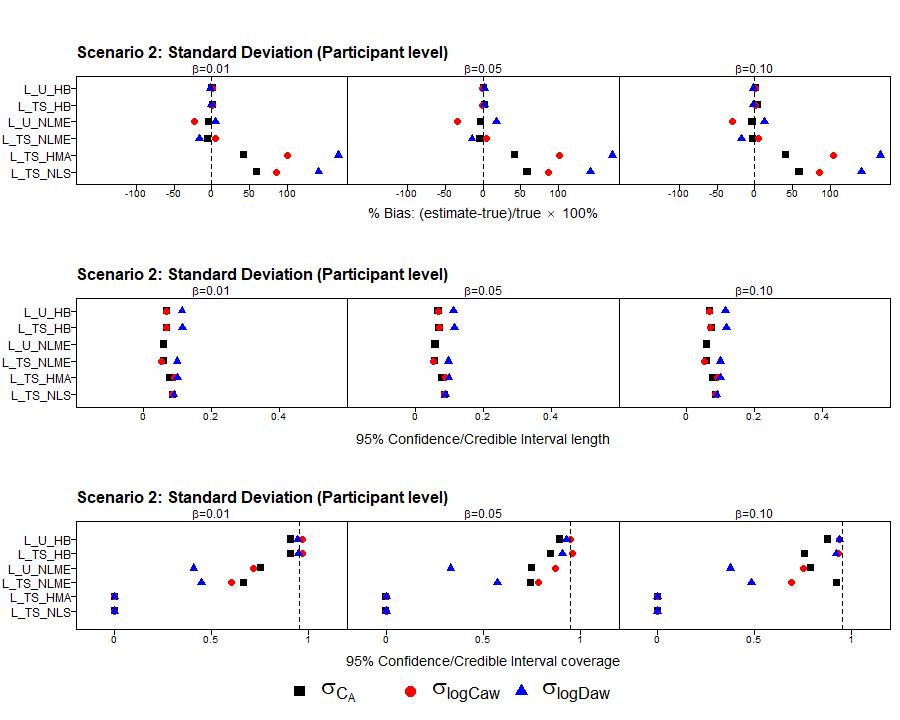 |

## C: Scenario 3

| C1: Estimated Associations β (Bias instead of % Bias) |
| --- |
| 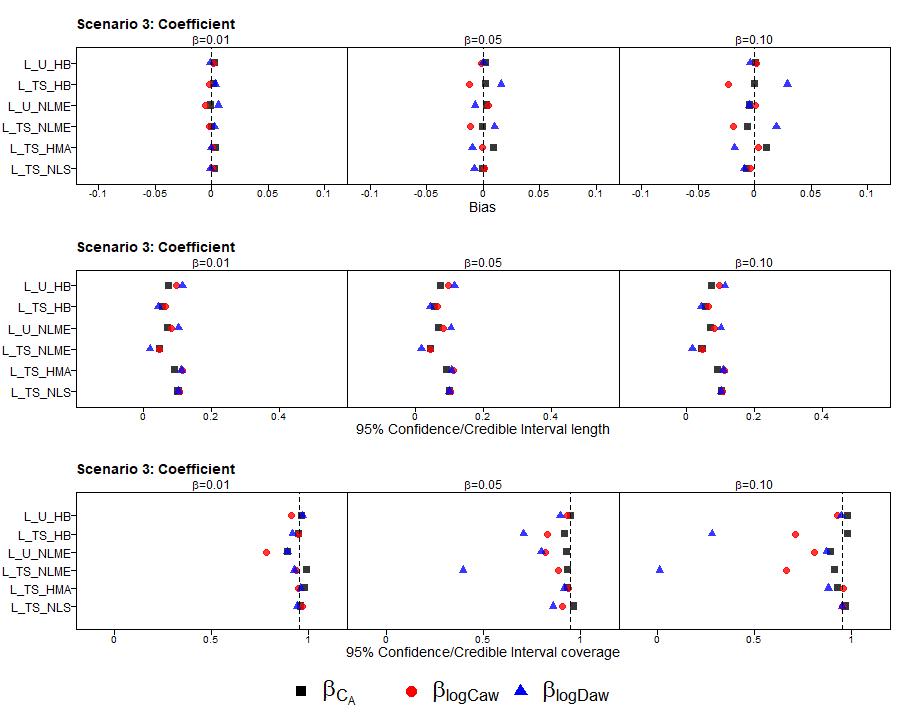 |
|  |
| 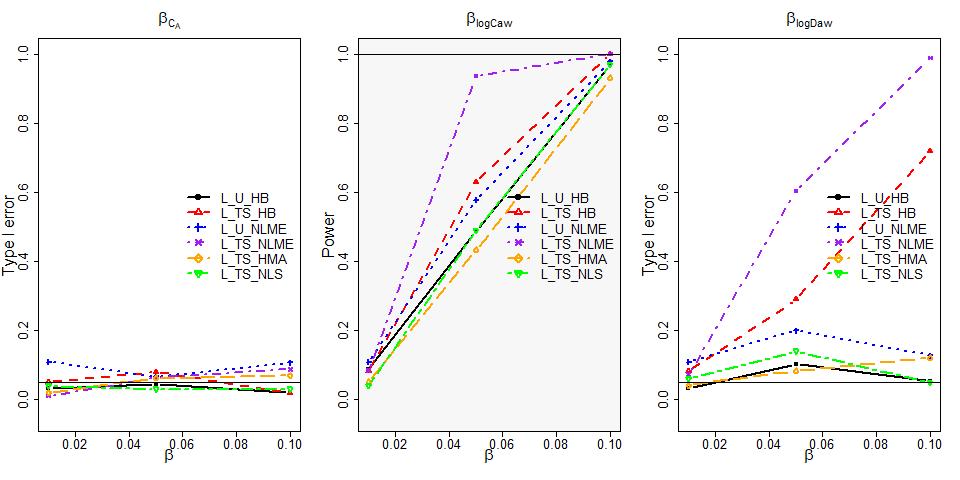 |

| C2: Population level mean ɑ |
| --- |
| 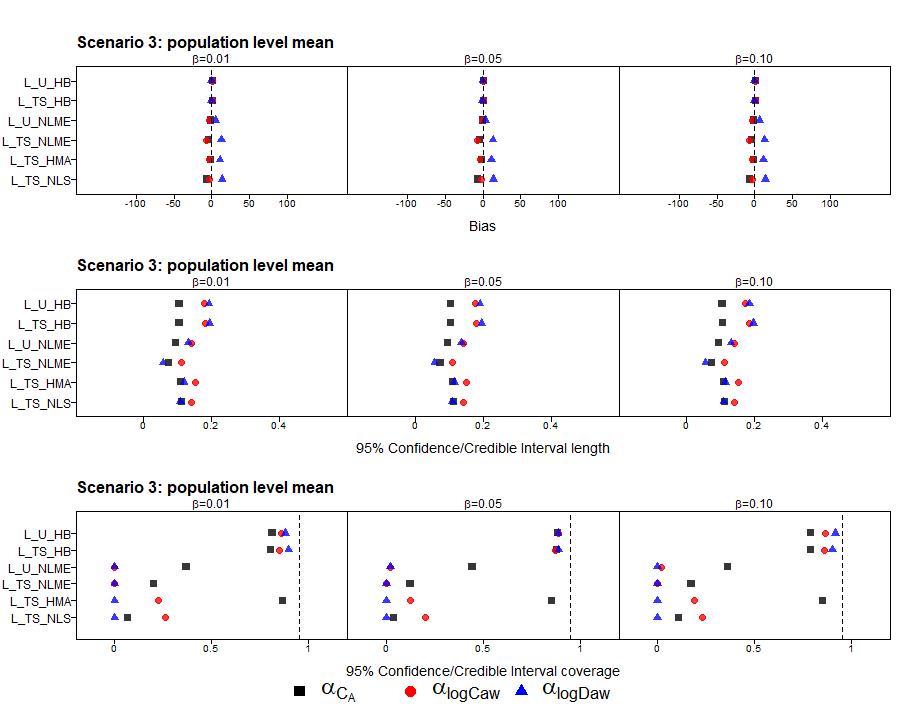 |

| C3: Participant level standard deviations τ |
| --- |
|  |


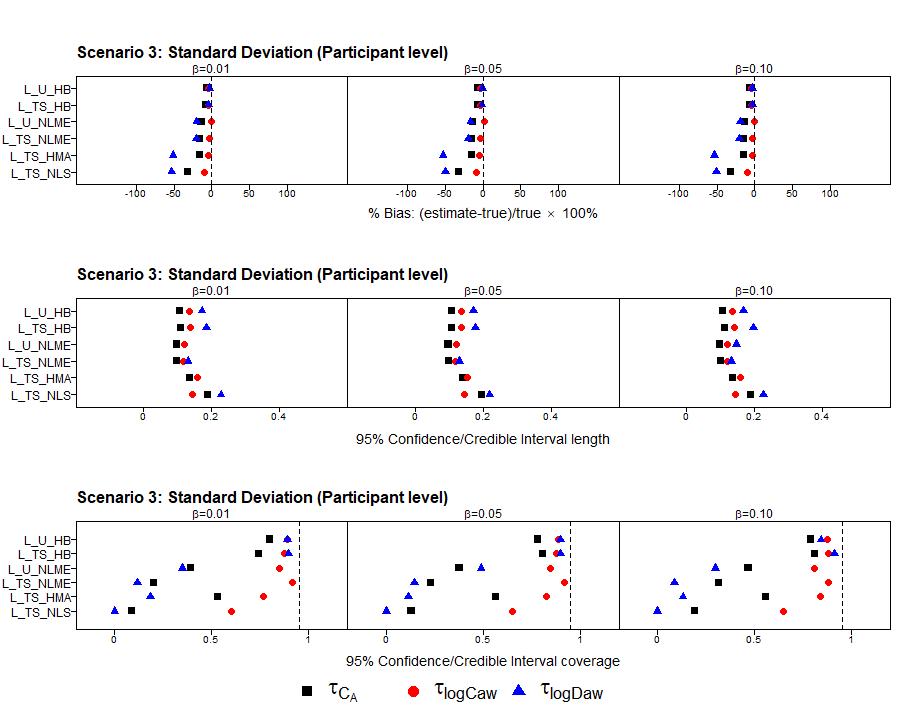


| C4: Participant level correlations ⍴ |
| --- |
| 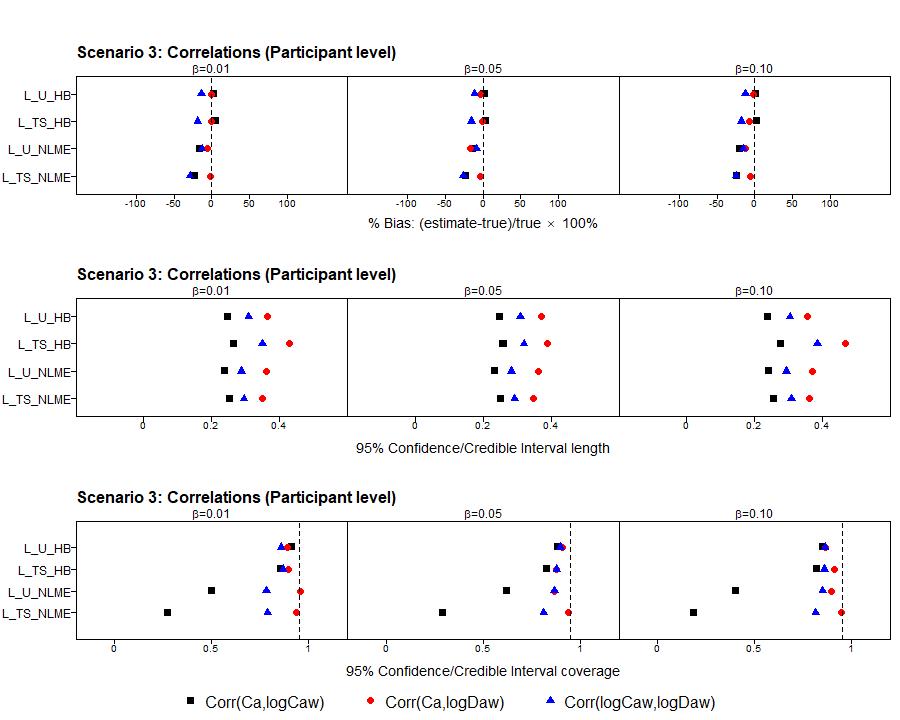 |

| C5: Visit level standard deviations σ |
| --- |
| 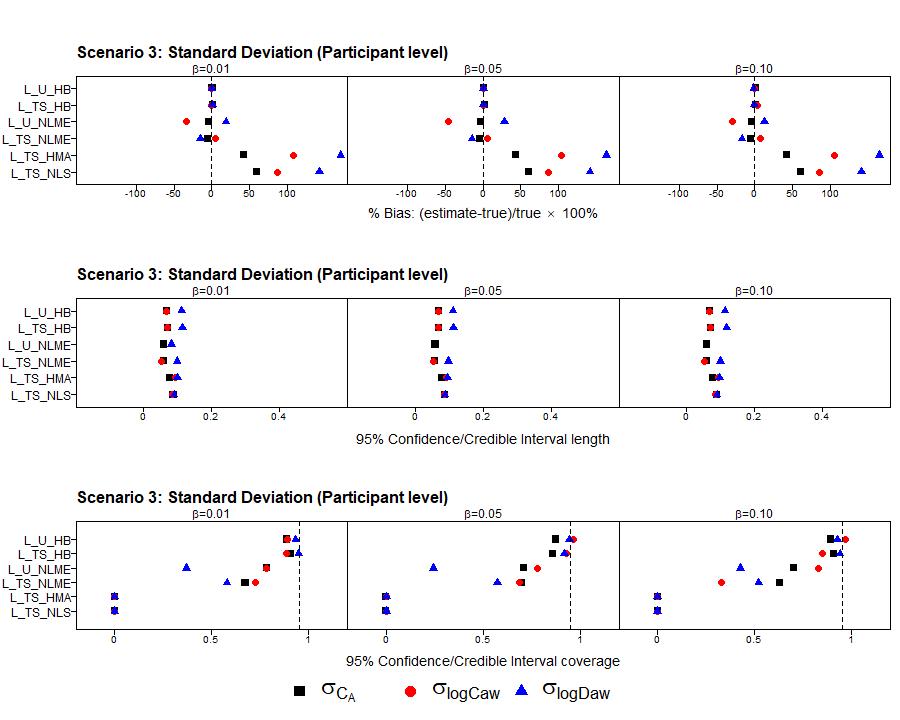 |

## D: Scenario 4

| D1: Estimated Associations β (Bias instead of % Bias) |
| --- |
| 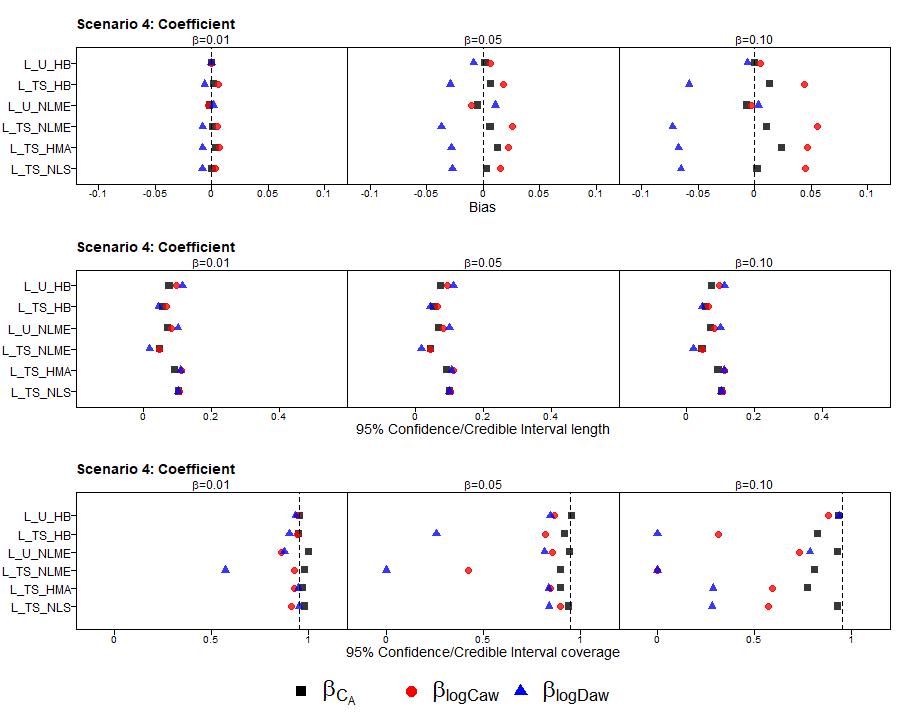 |
|  |
| 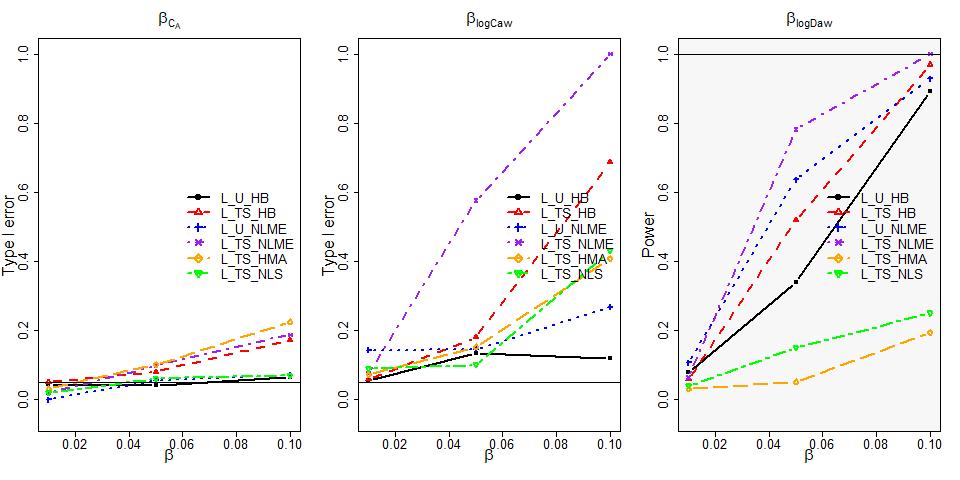 |

| D2: Population level mean ɑ |
| --- |
| 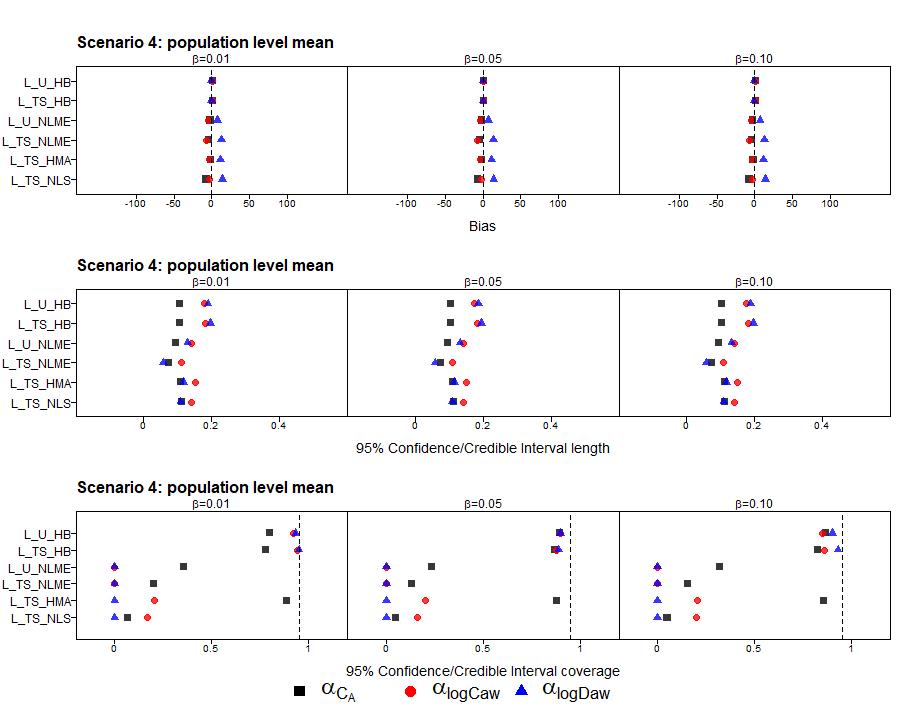 |

| D3: Participant level standard deviations τ |
| --- |
| 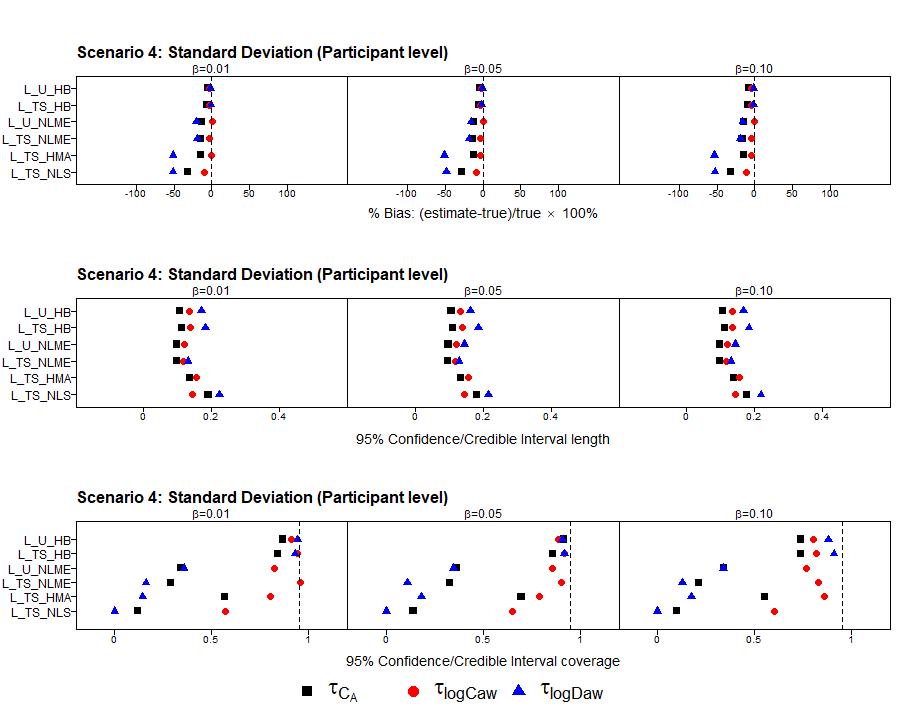 |

| D4: Participant level correlations ⍴ |
| --- |
| 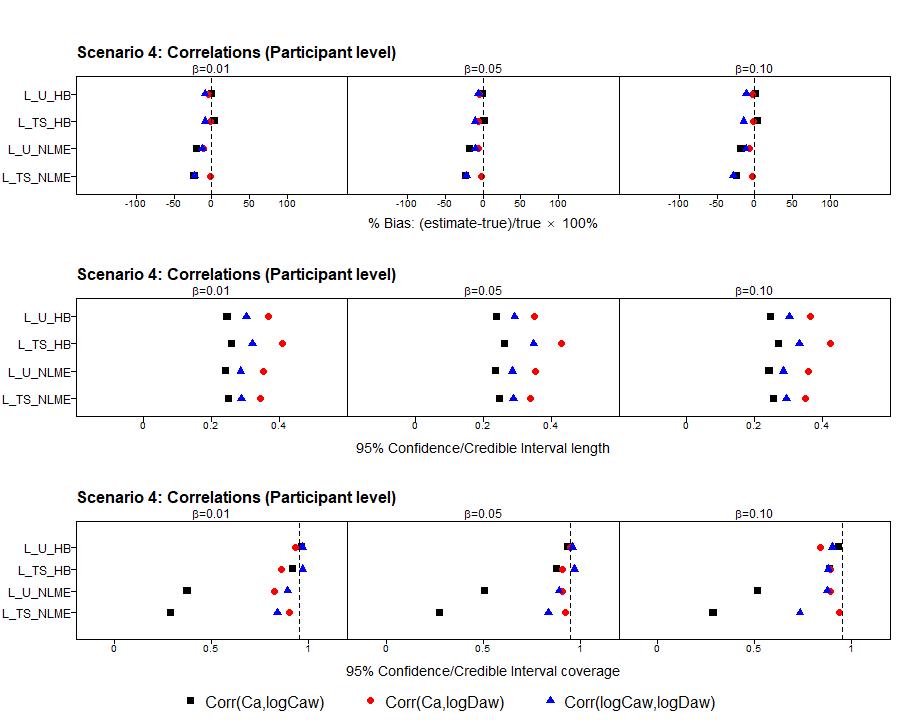 |

| D5: Visit level standard deviations σ |
| --- |
| 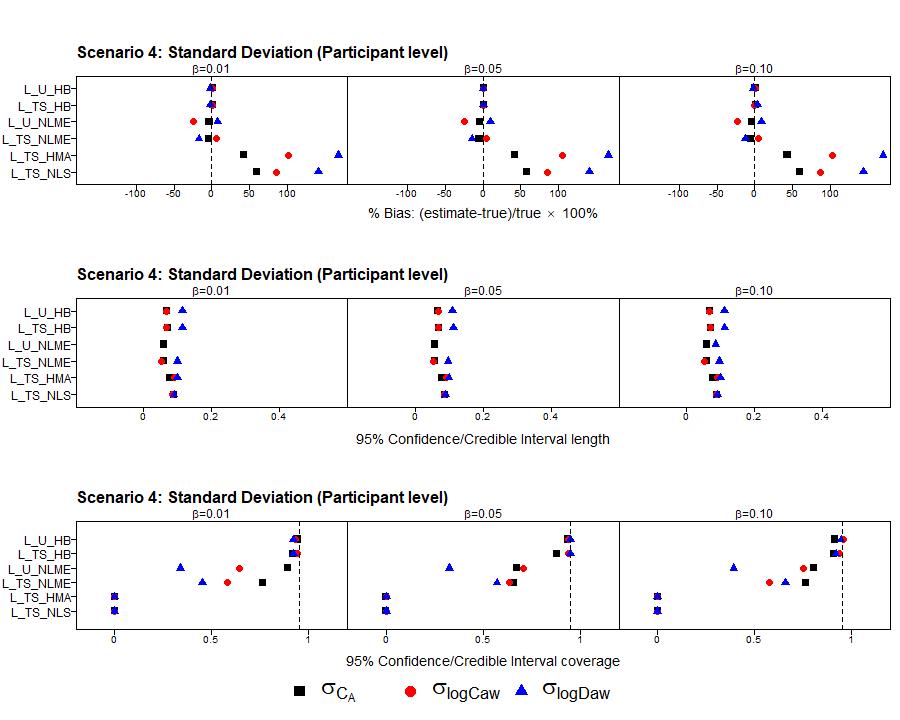 |

#

# Supplementary Figure 2: Summary on all converged datasets (44% - 64%) for Scenario 1 Estimated Associations β^^[[2]](#footnote-2)^^

Figure 2.a: The percentage bias (% bias), 95 % confidence interval length and the coverage of 95 % confidence intervals


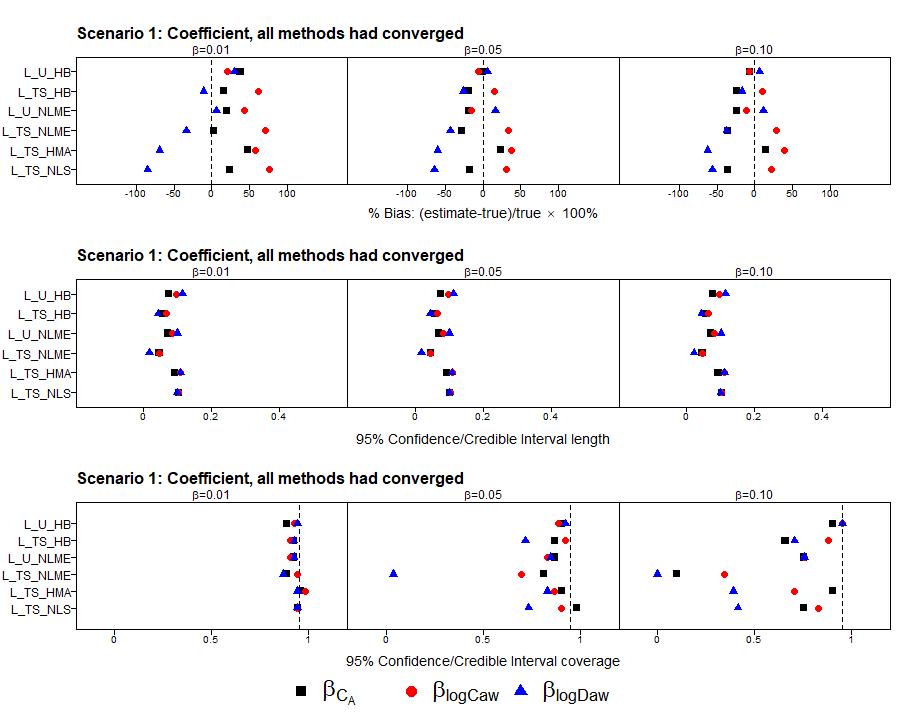


Figure 2.b: The power vs type I error rates:


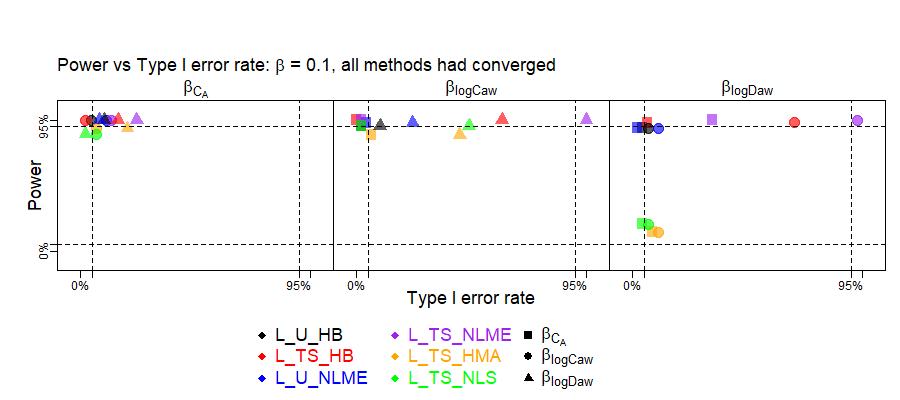


#

#

# Supplementary Figure 3: Constrain the HMA Stage I to be non-negative for NO parameters and ENO.

We previously used all estimations from Stage I for HMA analysis. However, if we use only non-negative estimations, the bias patterns were similar to other two-stage methods but at a cost of 26.4% drop rate on average.


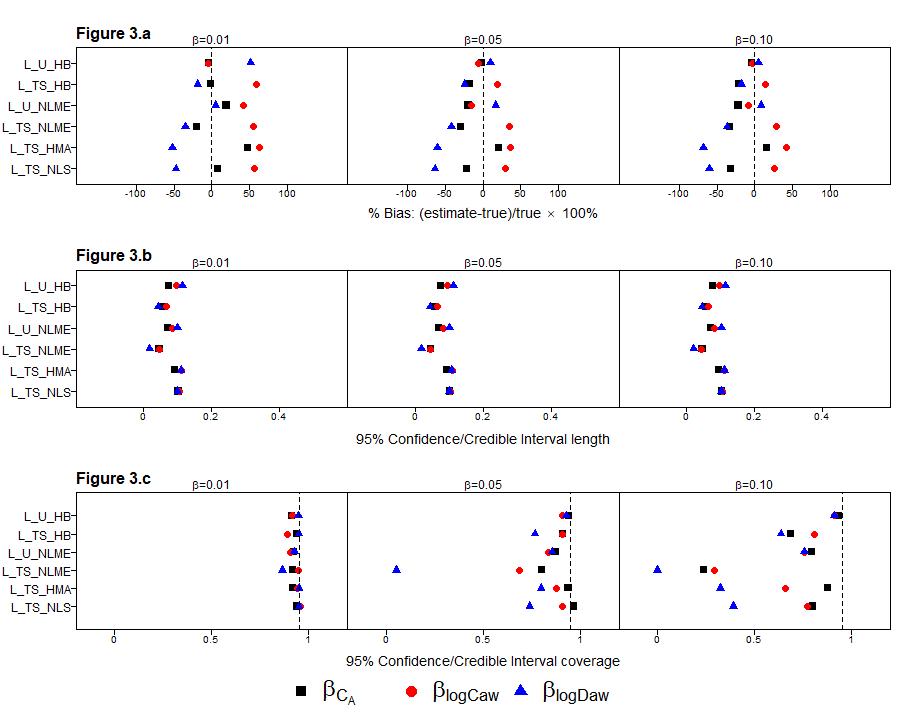


# Supplementary Table 3: CHS summary

The effects of height to the NO parameters among CHS study data were summarized in Table 3 in the form of Mean (95% CI). Significant associations were highlighted in yellow.

## Table 3.a: Mean (95% CI) for coefficient of standardized height

| Models | $\beta_{Ca}$ | $\beta_{logCaw}$ | $\beta_{logDaw}$ |
| --- | --- | --- | --- |
| L_U_HB | 0.079 (0.034, 0.125) | 0.158 (0.106, 0.212) | -0.106 (-0.171, -0.044) |
| L_TS_HB | 0.046 (0.014, 0.078) | 0.05 (0.027, 0.072) | 0.006 (-0.015, 0.028) |
| L_U_NLME | 0.092 (0.046, 0.138) | 0.149 (0.104, 0.194) | -0.104 (-0.157, -0.052) |
| L_TS_NLME | 0.038 (0.008, 0.067) | 0.057 (0.033, 0.081) | 0 (-0.021, 0.021) |
| L_TS_HMA | -0.019 (-0.098, 0.06) | 0.063 (0.009, 0.118) | -0.006 (-0.058, 0.046) |
| L_TS_NLS | -0.145 (-1.83, 1.54) | 0.08 (0.033, 0.127) | -0.016 (-0.057, 0.025) |

* On stage I, HMA: 401 failed; NLS: 542 failed

Full results

| parameter | L_U_HB | L_TS_HB | L_U_NLME | L_TS_NLME | L_TS_HMA | L_TS_NLS |
| --- | --- | --- | --- | --- | --- | --- |
| $ɑ_{Ca}$ | 1.943 (1.892, 1.995) | 1.939 (1.902, 1.976) | 1.732 (1.682, 1.782) | 1.726 (1.692, 1.761) | 1.914 (1.831, 1.997) | 1.006 (-0.704, 2.716) |
| $ɑ_{logCaw}$ | 4.412 (4.341, 4.485) | 4.392 (4.359, 4.426) | 4.011 (3.958, 4.064) | 4.004 (3.97, 4.039) | 4.152 (4.095, 4.209) | 4.129 (4.078, 4.18) |
| $ɑ_{logDaw}$ | 2.167 (2.086, 2.246) | 2.187 (2.146, 2.227) | 2.629 (2.569, 2.69) | 2.639 (2.604, 2.674) | 2.574 (2.521, 2.628) | 2.635 (2.592, 2.677) |
| $\tau_{Ca}$ | 0.507 (0.464, 0.557) | 0.528 (0.443, 0.599) | 0.629 (0.588, 0.674) | 0.458 (0.427, 0.491) | NA | NA |
| $\tau_{logCaw}$ | 0.709 (0.653, 0.77) | 0.696 (0.644, 0.754) | 0.52 (0.461, 0.586) | 0.522 (0.497, 0.55) | NA | NA |
| $\tau_{logDaw}$ | 0.861 (0.788, 0.942) | 0.843 (0.776, 0.927) | 0.702 (0.645, 0.764) | 0.546 (0.521, 0.573) | NA | NA |
| $\rho_{Ca,logCaw}$ | 0.816 (0.76, 0.883) | 0.849 (0.773, 0.905) | 0.834 (0.705, 0.91) | NA | NA | NA |
| $\rho_{Ca,logDaw}$ | -0.172 (-0.308, -0.044) | -0.208 (-0.338, -0.058) | -0.448 (-0.561, -0.318) | NA | NA | NA |
| $\rho_{logCaw,logDaw}$ | -0.706 (-0.759, -0.648) | -0.69 (-0.746, -0.631) | -0.571 (-0.633, -0.502) | NA | NA | NA |
| $\sigma_{Ca}$ | 0.659 (0.619, 0.698) | 0.656 (0.615, 0.699) | 0.369 (0.345, 0.394) | 0.468 (0.448, 0.488) | NA | NA |
| $\sigma_{logCaw}$ | 0.359 (0.331, 0.388) | 0.366 (0.339, 0.392) | 0.615 (0.573, 0.66) | 0.304 (0.291, 0.317) | NA | NA |
| $\sigma_{logDaw}$ | 0.37 (0.333, 0.408) | 0.367 (0.331, 0.402) | 0.355 (0.323, 0.39) | 0.243 (0.232, 0.253) | NA | NA |

1. Stage I convergence failure rate is the number of participant’s Stage I models that failed to converge divided by the total number of participants (500) [↑](#footnote-ref-1)
2. Instead of using all available converged results, we summarize those datasets converged by all methods. [↑](#footnote-ref-2)
